# Integration of PSAd and multiparametric MRI to forecast biopsy outcomes in biopsy-naïve patients with PSA 4~20 ng/ml

**DOI:** 10.3389/fonc.2024.1413953

**Published:** 2024-07-04

**Authors:** Lei Ren, Yanling Chen, Zixiong Liu, Guankai Huang, Weifeng Wang, Xu Yang, Baohua Bai, Yan Guo, Jian Ling, Xiaopeng Mao

**Affiliations:** ^1^ Department of Urology, The First Affiliated Hospital of Sun Yat-sen University, Sun Yat-sen University, Guangzhou, China; ^2^ Department of Radiology, The First Affiliated Hospital of Sun Yat-sen University, Sun Yat-sen University, Guangzhou, China; ^3^ Department of Urology, The Seventh Affiliated Hospital of Sun Yat-sen University, Sun Yat-sen University, Shenzhen, China; ^4^ Department of Urology, Hui Ya Hospital of The First Affiliated Hospital of Sun Yat-sen University, Sun Yat-sen University, Huizhou, China; ^5^ Department of Radiology, The Eastern Hospital of the First Affiliated Hospital of Sun Yat-sen University, Sun Yat-Sen University, Guangzhou, China

**Keywords:** prostate cancer, prostate biopsy, mpMRI, prostate-specific antigen density, PI-RADS

## Abstract

**Introduction:**

This study aims to investigate whether the transrectal ultrasound-guided combined biopsy (CB) improves the detection rates of prostate cancer (PCa) and clinically significant PCa (csPCa) in biopsy-naïve patients. We also aimed to compare the Prostate Imaging Reporting and Data System (PI-RADS v2.1) score, ADC values, and PSA density (PSAd) in predicting csPCa by the combined prostate biopsy.

**Methods:**

This retrospective and single-center study included 389 biopsy-naïve patients with PSA level 4~20 ng/ml, of whom 197 underwent prebiopsy mpMRI of the prostate. The mpMRI-based scores (PI-RADS v2.1 scores and ADC values) and clinical parameters were collected and evaluated by logistic regression analyses. Multivariable models based on the mpMRI-based scores and clinical parameters were developed by the logistic regression analyses to forecast biopsy outcomes of CB in biopsy-naïve patients. The ROC curves measured by the AUC values, calibration plots, and DCA were performed to assess multivariable models.

**Results:**

The CB can detect more csPCa compared with TRUSB (32.0% vs. 53%). The Spearman correlation revealed that Gleason scores of the prostate biopsy significantly correlated with PI-RADS scores and ADC values. The multivariate logistic regression confirmed that PI-RADS scores 4, 5, and prostate volume were important predictors of csPCa. The PI-RADS+ADC+PSAd (PAP) model had the highest AUCs of 0.913 for predicting csPCa in biopsy-naïve patients with PSA level 4~20 ng/ml. When the biopsy risk threshold of the PAP model was greater than or equal to 0.10, 51% of patients could avoid an unnecessary biopsy, and only 5% of patients with csPCa were missed.

**Conclusion:**

The prebiopsy mpMRI and the combined prostate biopsy have a high CDR of csPCa in biopsy-naïve patients. A multivariable model based on the mpMRI-based scores and PSAd could provide a reference for clinicians in forecasting biopsy outcomes in biopsy-naïve patients with PSA 4~20 ng/ml and make a more comprehensive assessment during the decision-making of the prostate biopsy.

## 1 Introduction

Men with an elevated prostate-specific antigen (PSA > 4 ng/ml) or an abnormal digital rectal examination are usually recommended to undergo the transrectal ultrasound-guided systematic prostate biopsy (TRUSB) for diagnosing prostate cancer (PCa) (1). However, this often leads to the underdiagnosis of clinically significant PCa (csPCa) and overdiagnosis of low-risk PCa with low Gleason scores (2, 3). Over the past two decades, multiparametric magnetic resonance imaging (mpMRI) has significantly improved the diagnosis rates of PCa, especially csPCa, and targeted biopsy of suspicious lesions identified by mpMRI (MRI-TB) has been proven by many clinical studies to detect more csPCa and less clinically insignificant PCa (icsPCa) compared with TRUSB (3–5). The combined biopsy of prostate cancer (targeted plus systematic biopsies) also showed high detection of csPCa compared with TRUSB (6, 7). However, there is little knowledge about the effectiveness of the combined biopsy for biopsy-naïve men with PSA < 20 ng/ml in China.

According to many guidelines, mpMRI is highly recommended before the biopsy of every patient with clinically suspicious PCa (8, 9). In 2019, the Prostate Imaging Reporting and Data System (PI-RADS) was updated to version 2.1 by the European Society of Urogenital Radiology (ESUR), the American College of Radiology, and other institutes (10). Biopsy-naïve men with PI-RADS scores ≥ 3 are always recommended for the MRI-targeted biopsy (MRI-TB) (10, 11). Diffusion-weighted imaging (DWI) is one of the most significant parts of mpMRI, commonly measured by the apparent diffusion coefficient (ADC) value. Previous studies have revealed that ADC value is negatively correlated with Gleason score (GS), indicating it may contribute to clinical diagnosis (prediction of prostate biopsy outcome) and management (12–14). In recent years, micro-ultrasound (Micro US) is a novel high-resolution ultrasound technology in PCa detection that can be used to indicate suspicious lesions in the prostate, guide prostate biopsy, and provide real-time visualization during the biopsy (15, 16). Previous studies have revealed that Micro US as a potentially cost and time-saving new technology showed comparable accuracy to mpMRI in guidance of prostate biopsy (15). Elevated PSA levels in the diagnosis of csPCa show a high sensitivity but a relatively low specificity. The PSA density (PSAd) may improve the diagnostic value of PSA and reduce the likelihood of false positive outcomes (17). A biopsy strategy combining PI-RADS score and PSAd has been proven to predict the biopsy outcomes and safely reduce unnecessary biopsies in biopsy-naïve men (18). Therefore, the combination of mpMRI-based radiomics and clinical parameters could assist clinicians in avoiding unnecessary prostate biopsy.

In our study, we evaluated whether the prostate CB can find more PCa and csPCa compared with TRUSB and developed a multivariable model based on the mpMRI-based scores and clinical parameters to provide a reference for clinicians in forecasting biopsy outcomes in biopsy-naïve patients with PSA 4~20 ng/ml and making a more comprehensive assessment during the decision-making of the prostate biopsy.

## 2 Methods

### 2.1 Patient population

This was a retrospective and single-center study approved by the ethics committee of the First Affiliated Hospital of Sun Yat-sen University (2023–439), which waved off the requirement for informed consent. From July 2016 to May 2022, men with elevated PSA levels (4 to 20 ng/ml) who had never undergone a prostate biopsy previously were included in our study. The details of the inclusion criteria are as follows: (1) patients with elevated PSA level within the previous 3 months before the prostate biopsy; (2) patients who underwent their first transrectal prostate biopsy at our institution; (3) if patients underwent prebiopsy MRI of the prostate within the previous 3 months, it must contain the T2WI and DWI, and PI-RADS v2.1 score ≥ 2; and (4) complete clinical information could be obtained, which included age, total PSA (tPSA) level, biopsy outcomes, and biopsy cores. The exclusion criteria are as follows: (1) patients had undergone prior prostate surgery or biopsy; (2) androgen deprivation therapy, pelvic radiotherapy, and chemotherapy of PCa; (3) diagnosed with a tumor other than PCa; (4) imaging examination suggested distant metastasis or lymph node metastasis. In total, 389 patients were included in our study, and there were 197 patients who underwent prebiopsy mpMRI of the prostate and 82 patients with PI-RADS v2.1 scores = 2.

### 2.2 mpMRI

Patients underwent mpMRI before the prostate biopsy using a 3.0-T scanner (Signa Pioneer, GE, Milwaukee, WI, United States) with a standard spine array coil and 32-channel body array coil. The standard mpMRI protocol included triaxial (axial, coronal, and sagittal) T1-weighted imaging, T2–T2-weighted imaging, and diffusion-weighted imaging (DWI). The corresponding apparent diffusion coefficient (ADC) maps were generated. All mpMRI imaging was reviewed by two professional radiologists with 10 years of experience in reporting prostate mpMRI, who were blind to the clinical information of patients. The PI-RADS v2.1 was used in our study to assess the mpMRI results (10). In DWI imaging of prostate mpMRI, the suspicious region of PCa was circled with the most cross-sectional area of the PCa (region of interest, ROI), which did not include the normal area (13). Next, three small circular regions that did not overlap with each other were drawn in the ROI, and ADC values in these regions were measured respectively. Finally, the average of the three regions’ ADC values was selected for further analysis. Prostate volume (PV) was measured based on mpMRI with the following formulation: (maximum anteroposterior diameter) × (maximum transverse diameter) × (maximum longitudinal diameter) × 0.52 (10). When the PI-RADS score of prostate mpMRI was inconsistent and the difference of ADC and/or prostate volume was hugely evaluated by both two radiologists, the third more experienced radiologist stepped in and discussed with them to determine the final results.

### 2.3 Prostate biopsy

All prostate biopsies were performed under the guidance of transrectal ultrasound (Esaote, Mylab 9) in our institute by urologists with 5-year experience in prostate biopsy using a BARD ejection biopsy gun and an 18-G needle with an 18~22-mm depth of biopsy. All patients were required to take levofloxacin tablets orally (500 mg/day) 4 days from 1 day before the biopsy as prophylactic antibiotics and were also given a clean enema with 500 ml normal saline 2 h before the biopsy. Patients with PI-RADS scores ≥ 3 underwent the transrectal ultrasound-guided combined biopsy (CB) with 10 to 12 cores, which comprised targeted biopsy (TB) and systematic biopsy (SB). The urologists utilized the cognitive fusion approach (matching the lesions on mpMRI and the real-time images of lesions under transrectal ultrasound) to perform TB (4). Two cores of TB were taken from the suspicious lesions with PI-RADS scores ≥ 3. If there were more than two suspicious lesions on mpMRI, two cores were obtained from the highest scores, and one core was obtained from the lowest score. Next, the SB with six to eight cores was done in the nonsuspicious lesions. Patients with PI-RADS scores = 2 or who did not undergo mpMRI had 10 to 12 cores of SB from the peripheral zone of the prostate at the apex, mid-gland, and base (6). Each core of the biopsy was labeled individually. A Gleason score ≥ 3 + 4 was defined as csPCa, whereas others were defined as clinically insignificant PCa (icsPCa) (19).

### 2.4 Statistical analysis

A total of 197 patients who underwent prostate mpMRI belonged to the CB group, while 192 patients underwent SB and belonged to the SB group. Meanwhile, 197 patients in the CB group were included in the study of the model establishment. The clinical information of these patients included age, total PSA, PV, PSA density (PSAd, the ratio of tPSA to PV, ng/ml^2^), and Gleason scores. mpMRI-based scores data included PI-RADS score and ADC values. The *t-*test was utilized to compare continuous variables (age and ADC values) between the two patient groups, while the chi-squared test or Fisher’s test was used to compare categorical variables (PI-RADS score). Due to the abnormal distribution of PSA, PSAd, and PV, the nonparametric Wilcoxon test was utilized to compare these variables between the two patient groups. To compare the difference in the number of PCa and cancer detection rate (CDR) between the CB group and SB group, the chi-squared test was performed. Due to dichotomous outcomes (PCa or noncancer) in our model, the continuous variables of ADC values were transformed into categorical variables according to cut-off values. Univariate and multivariate logistic regression analyses were performed to identify the significant predictors of PCa and csPCa. In order to better benefit the clinical application, we constructed three multivariable models to evaluate the possibility of PCa and csPCa at the prostate biopsy by logistic regression model analysis: the ADC value and PSAd model; the PI-RADS and PSAd model; the PI-RADS, ADC value, and PSAd model. We randomly separated all 197 patients into two validation cohorts in a 6:4 ratio: validation cohort 1 and validation cohort 2. The receiver operating characteristic (ROC) curves measured by the area under the curve (AUC) values were performed to evaluate the predictive accuracy of clinical information, mpMRI-based scores, and three models in forecasting the PCa and csPCa at biopsy and compare the discriminatory ability of these three models. Decision curve analysis (DCA) was performed to evaluate the clinical application of alternative diagnostic strategies (biopsy strategies) by the “rmda” R package (20). Spearman correlation analysis was performed to assess the relationship between clinical information, PI-RADS, ADC value, and Gleason scores. SPSS version 25 (IBM SPSS Statistics for Windows, version 25.0 Inc., Chicago, IL, USA) was used to perform all statistical analyses. Statistical significance was defined as two-sided *p* < 0.05 when no special description exists. A nomogram integrating the PI-RADS score, ADC value, and clinical characteristics (PSA, PV, and age) was established to further augment the clinical implementation of PI-RADS score the by the “rms”, “foreign”, and “survival” R packages. R software (ver.4.3.3, the R foundation for statistical computing, Vienna, Austria) was used to analyze data and visualize the results.

## 3 Results

From July 2016 to May 2022, 389 men were enrolled in our study. A total of 197 of 389 patients who underwent the prostate mpMRI were included in the establishment of multivariable models, and 82 patients had no suspicious lesions on mpMRI (PI-RADS scores = 2) who underwent the SB, while 115 patients showed a result on mpMRI that suspicious lesions of PCa who underwent the CB. Next, 192 patients who did not undergo the prostate mpMRI had the SB. Therefore, a total of 197 men were divided into the CB group, while 192 men were in the SB group.

### 3.1 Comparison of clinical features and CDRs in CB and SB groups

The patient characteristics in the CB and SB groups are shown in **Table 1**. The median (interquartile range, IQR) age and PSA in the CB and SB groups were 68.0 (63–73) and 67.3 (62–74) years, or 11.15 (7.64–14.98) and 12.50 (9.46–15.75) ng/ml, respectively. For CDRs of prostate cancer (PCa), 88 men (44.7%) were detected with PCa in the CB group, and 79 men (41.1%) were detected with PCa in the SB group. As for clinically significant PCa (csPCa), there was also a difference in CDRs between the two groups, with 63 men (32.0%) diagnosed with csPCa in the CB group and 53 men (27.6%) diagnosed with csPCa in the SB group. The Gleason scores of the biopsy in the two groups are also shown in **Table 1**. Moreover, patients with PI-RADS scores = 2 in the CB group underwent the SB; therefore, we compared the CDR between the CB and SB methods. We found that the CB could detect more csPCa compared to the SB (**Table 1**).

**Table 1:** Comparison of basic characteristics and cancer detection of biopsy results between CB and SB groups.

Features	CB group (*N* = 197)	SB group (*N* = 192)
Age (years)*	68.0 (63.0–73.0)	67.3 (62.0–74.0)
PSA (ng/ml)*	11.15 (7.64–14.98)	12.50 (9.46–15.75)
Biopsy outcome (no)^†^		
BPH	106	105
PIN	3	8
PCa	88 (44.7%)	79 (41.1%)
csPCa	63 (32.0%)	53 (27.6%)
icsPCa	25 (12.7%)	26 (13.5%)
Gleason score		
6	25	26
7	35	25
8	19	17
9	8	10
10	1	1

* Data refer to medians, with interquartile ranges in parentheses (IQR).

† Data refer to the number of patients.

PCa, prostate cancer; csPCa, clinically significant prostate cancer; icsPCa, clinically insignificant prostate cancer; CB group, combined (targeted and systematic) biopsy; SB group, systematic biopsy.

### 3.2 Patient basic characteristics in the model establishment

The basic characteristics of 197 patients in the multivariable models are shown in **Table 2**; the median (interquartile range (IQR)) age, PSA, prostate volume (PV), and ADC values were 68.0 (63–73) years, 11.15 (7.64–14.98) ng/ml, 49.70 (30.90–59.45) ml, 0.33 (0.18–0.47) ng/ml^2^, and 1,117 (807–1,259). As for biopsy outcomes, 88 patients (45%) were diagnosed with PCa, of which 25 (13%) were icsPCa and 63 (32%) were csPCa (**Table 3**). Compared with noncancer patients, men with PCa were marginally older, had higher PSAd, and had smaller PV. Men with PCa also had higher PI-RADS scores and lower ADC values in mpMRI (**Table 4**). Additionally, men with csPCa were also older, had higher PSA levels and PSAd, and had a smaller PV compared to non-csPCa men (**Supplementary Table S1**). The cut-off value of PSAd for diagnosing csPCa in our study was 0.23 ng/ml^2^ with a PI-RADS score ≥ 2. Furthermore, when the PI-RADS score was ≥ 3, the cut-off value of PSAd for diagnosing csPCa was 0.33 ng/ml^2^. The Spearman’s correlation analysis revealed that Gleason scores of the prostate biopsy significantly correlated with PI-RADS scores (*r* = 0.479, *p* < 0.001) and ADC values (*r* = −0.364, *p* < 0.001) (**Supplementary Table S2**).

**Table 2:** Characteristics of 197 patients with prostate mpMRI.

Features	Value
Median (IQR)	
Age (years)	68.0 (63–73)
PSA (ng/ml)	11.15 (7.64–14.98)
Prostate volume (ml)	49.70 (30.90–59.45)
PSAd (ng/ml/ml)	0.33 (0.18–0.47)
mpMRI-based radiomics	
PI-RADS (v2.1)^†^	
2	82
3	32
4	33
5	50
ADC	1,117 (807–1,259)

† Data refer to the number of patients.

IQR, interquartile ranges in parentheses; PSA, prostate-specific antigen; PSAd, PSA density; mpMRI, multiparametric MRI; PI-RADS, Prostate Imaging Reporting and Data System; ADC, apparent diffusion coefficient.

**Table 3:** Outcomes of the prostate biopsy in 197 patients with prostate mpMRI.

Outcomes	*N* (%)
Atypical hyperplasia	9 (4.5)
BPH	97 (49)
PIN	3 (1.5)
Prostate cancer	88 (45)
Clinically insignificant	25 (13)
Clinically significant	63 (32)

BPH, benign prostatic hyperplasia; PIN, prostatic intraepithelial neoplasia.

**Table 4:** Clinical features of PCa patients and noncancer patients with prostate mpMRI.

Features	PCa	No cancer	P value
Age (years)*	69.50 (64.0–75.0)	66.60 (63.0–71.0)	0.005
PSA (ng/ml)*	11.98 (8.10–15.77)	10.49 (7.33–13.45)	0.101
Prostate volume (cm^3^)*	37.90 (26.4–43.8)	59.20 (38.6–76.2)	< 0.001
PSAd (ng/ml^2^)^†^	0.37 (0.23–0.50)	0.30 (0.15–0.43)	< 0.001
mpMRI			
PI-RADS (v2.0)^†^			
2	6	76	< 0.001
3	11	21	< 0.001
4	28	5	< 0.001
5	43	7	< 0.001
ADC value*	908 (685–1,032)	1,287 (1,083–1,347)	< 0.001
Gleason score			
6	25		
7	35		
8	19		
9	8		
10	1		

* Data refer to medians, with interquartile ranges in parentheses (IQR).

† Data refer to the number of patients.

ADC, apparent diffusion coefficient (measured in DWI).

Based on the cut-off value and IQR of PSAd and ADC values, 197 patients were stratified with different subgroups, and the biopsy outcomes were divided into three clusters (icsPCa; csPCa; no cancer) (**Supplementary Figure S1**). When PSAd ≥ 0.18 ng/ml, ADC value < 1300, or PI-RADS score ≥ 3, the PCa (especially csPCa) was considered, and prostate biopsy is recommended. The csPCa proportion was 21% (16/77), 50% (14/28), and 77% (23/28) when PSAd was 0.18~0.33, 0.33~0.47, and > 0.47, respectively. When ADC value was 1,100~1,300, 800~1,100, and < 800, the proportion of csPCa was 10% (6/58), 31% (17/54), and 75% (36/48), respectively. For PI-RADS = 3, 4, or 5, the csPCa proportion was 6% (2/32), 63.6% (21/33), and 74% (37/50), respectively.

### 3.3 Establishment of multivariable models

The univariate logistic regression analysis revealed that clinical parameters (age, PSA, PV, and PSAd) and mpMRI-based scores (PI-RADS score and ADC value) were all important predictors of PCa and csPCa (**Table 5**). The multivariate logistic regression analysis demonstrated PSAd, PI-RADS score, and ADC value as independent predictors for PCa (**Table 6**). Meanwhile, the PI-RADS score and ADC value were all independent predictors for csPCa.

**Table 5:** The univariate logistic regression analysis to identify the significant predictors of PCa and csPCa.

Features	Univariate analysis			
	All PCa		CsPCa	
	OR (95% CI)	*p*-value	OR (95% CI)	*p*-value
**Age (years)**		0.006		0.003
≤ 65		Ref		Ref
> 65, < 75	1.783 (0.932–3.410)	0.081	1.714 (0.830–3.536)	0.145
≥ 75	3.853 (1.666–8.909)	0.002	4.392 (1.855–10.398)	0.001
**PSA (ng/ml)**	1.083 (1.014–1.157)	0.018	1.077 (1.005–1.154)	0.037
**PV (ml)**	0.960 (0.945–0.977)	< 0.001	0.965 (0.948–0.982)	< 0.001
**PSAd (ng/ml^2^)**	2,735.895 (190.455–39,301.355)	< 0.001	327.522 (38.662–2,774.558)	< 0.001
**PI-RADS**		< 0.001		< 0.001
2		Ref		Ref
3	6.635 (2.196–20.050)	0.001	1.756 (0.279–11.031)	0.548
4	70.933 (20.051–250.931)	< 0.001	46.083 (11.904–178.399)	< 0.001
5	77.810 (24.569–246.422)	< 0.001	74.949 (20.128–279.081)	< 0.001
**ADC**		< 0.001		< 0.001
≤ 850		Ref		Ref
> 850, ≤ 1,000	0.145 (0.045–0.465)	0.001	0.182 (0.062–0.531)	0.002
> 1,000, ≤ 1,200	0.070 (0.026–0.190)	< 0.001	0.035 (0.011–0.112)	< 0.001
> 1,200	0.017 (0.006–0.050)	< 0.001	0.047 (0.018–0.119)	<0.001

N, number of men; OR, odds ratio; Ref, reference; csPCa, clinically significant PCa; PSA, prostate-specific antigen; PV, prostate volume; PSAd, PSA density; ADC, apparent diffusion coefficient.

**Table 6:** The multivariate logistic regression analysis to identify the significant predictors of PCa and csPCa.

Features	Multivariate analysis			
	All PCa		CsPCa	
	OR (95% CI)	*p*-value	OR (95% CI)	*p*-value
**PSAd (ng/ml^2^)**	140.162 (5.527–3,554.239)	0.003	14.270 (0.884–230.276)	0.061
**PI-RADS**		< 0.001		< 0.001
2		Ref		Ref
3	5.067 (1.481–17.343)	0.010	3.085 (0.435–21.895)	0.260
4	29.353 (6.757–127.513)	< 0.001	63.673 (8.533–475.141)	< 0.001
5	19.820 (4.321–91.002)	< 0.001	88.190 (11.067–702.755)	< 0.001
**ADC**		0.048		0.058
≤ 850		Ref		Ref
> 850, ≤ 1,000	0.180 (0.043–0.758)	0.019	0.265 (0.076–0.918)	0.036
>1,000, ≤1,200	0.374 (0.083–1.691)	0.201	0.341 (0.073–1.597)	0.172
>1,200	0.176 (0.038–0.810)	0.026	1.793 (0.268–11.269)	0.561

OR, odds ratio; Ref, reference; csPCa, clinically significant PCa; PSAd, PSA density; ADC, apparent diffusion coefficient.

According to the univariate logistic regression analysis, we selected the important predictors of PCa and csPCa for the establishment of multivariable models. We excluded PSA and PV from the establishment of models in order to avoid multicollinearity. Therefore, PSAd, PI-RADS score, and ADC value were involved in the establishment of multivariable models based on the binary logistic regression analysis. Based on these three factors, we constructed three models to predict the biopsy outcome by the multivariate logistic regression analysis: the ADC+PSAd (AP) model, the PI-RADS+PSAd (PP) model, and the PI-RADS+ADC+PSAd (PAP) model.

### 3.4 Assessment of multivariable models

**Figure 1:**
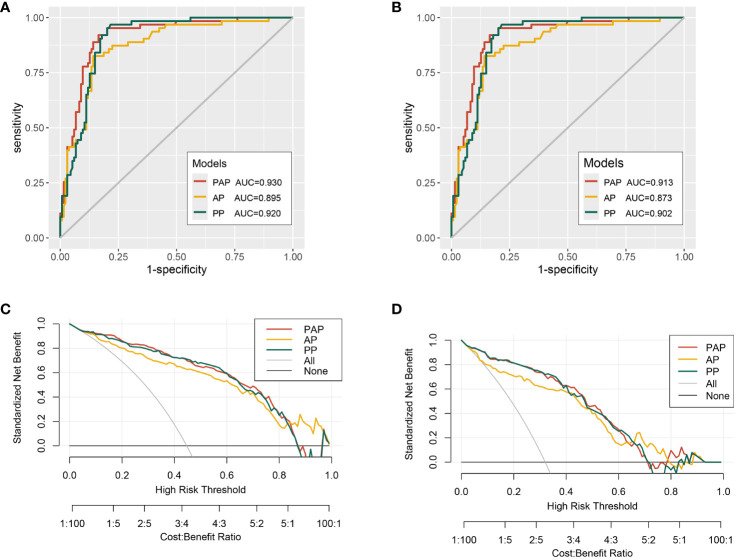
The receiver operating characteristic (ROC) curves and decision curve analysis (DCA) of the multivariable models. **(A)** ROC curves for the detection of PCa. **(B)** ROC curves for the detection of csPCa. **(C)** The multivariable models for predicting PCa. **(D)** The multivariable models for predicting csPCa.

ROC curve analysis showed the discriminatory ability of three models measured by AUC values. In predicting the PCa of the biopsy, the AUC was 0.895 for the AP model, 0.920 for the PP model, and 0.930 for the PAP model (**Figure 1A**). In predicting the csPCa of the biopsy, the AUC was 0.873 for the AP model, 0.902 for the PA model, and 0.913 for the PAP model (**Figure 1B**). The results of the ROC curve analysis revealed that the PAP model had the highest AUCs than the other two models, indicating its great discriminatory and predictive ability in predicting the PCa and csPCa of the biopsy outcome. The decision curve analysis revealed that the PAP model showed a significantly higher net benefit in avoiding unnecessary biopsy of PCa than the other models at clinical risk thresholds of 16%–35% and 62%–78% (**Figure 1C**). As shown in **Figure 1D**, the PAP model had a similar net benefit with the PP model at the 5%–40% clinical risk threshold, and at the 500%–60% clinical risk threshold, the PAP model had a significantly higher net benefit in avoiding unnecessary biopsy of csPCa than the other models.

**Figure 2:**
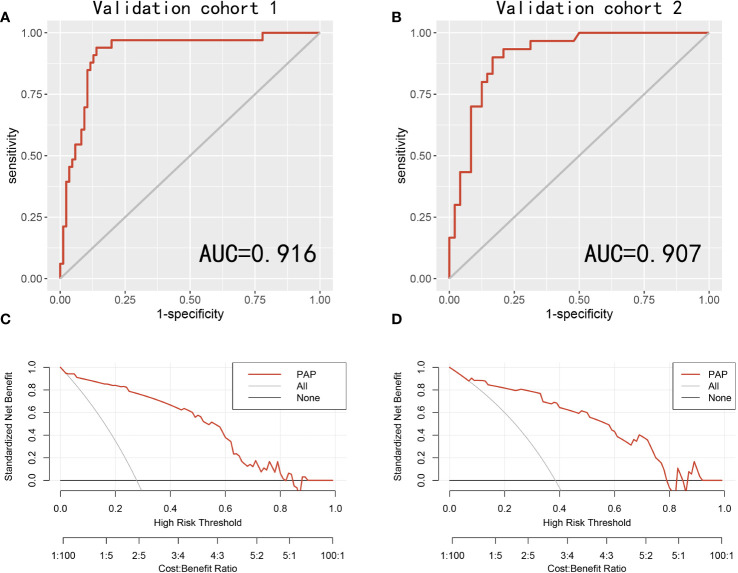
The receiver operating characteristic (ROC) curves and decision curve analysis (DCA) of the PAP models in predicting csPCa in two validation cohorts. **(A)** ROC curves for the detection of csPCa in the validation cohort 1. **(B)** ROC curves for the detection of csPCa in the validation cohort 2. **(C)** The PAP models for predicting csPCa in the validation cohort 1. **(D)** The PAP models for predicting csPCa in the validation cohort 2.

To further assess the PAP model in predicting the csPCa, we randomly separated all 197 patients into two validation cohorts in a 6:4 ratio: validation cohort 1 (*n* = 118) and validation cohort 2 (*n* = 79). We found that the AUCs of the validation cohorts 1 and 2 were 0.916 and 0.907, respectively (**Figures 2A, B**). Moreover, the decision curve analysis revealed that the PAP model had a significantly higher net benefit in avoiding unnecessary biopsy of csPCa in the two validation cohorts (**Figures 2C, D**).

**Supplementary Table S3** shows the sensitivity, specificity, positive predictive value (PPV), and negative predictive value (NPV) of the different biopsy strategies by the PAP model and PI-RADS scores in avoiding unnecessary biopsy. When the biopsy risk threshold was greater than or equal to 0.10, 51% of patients could avoid unnecessary biopsy, and only 3% of patients with csPCa were missed. The sensitivity, specificity, PPV, and NPV of this biopsy risk threshold were 0.95, 0.75, 0.65, and 0.97, respectively. In our present study, a PSAd cut-off value of 0.23 was established. When this value (PSAd ≥ 0.23 ng/ml^2^) was considered to define csPCa, the biopsy risk threshold demonstrated a sensitivity of 0.75, a specificity of 0.58, a PPV of 0.46, and an NPV of 0.83.

### 3.5 Construction of the nomogram

**Figure 3:**
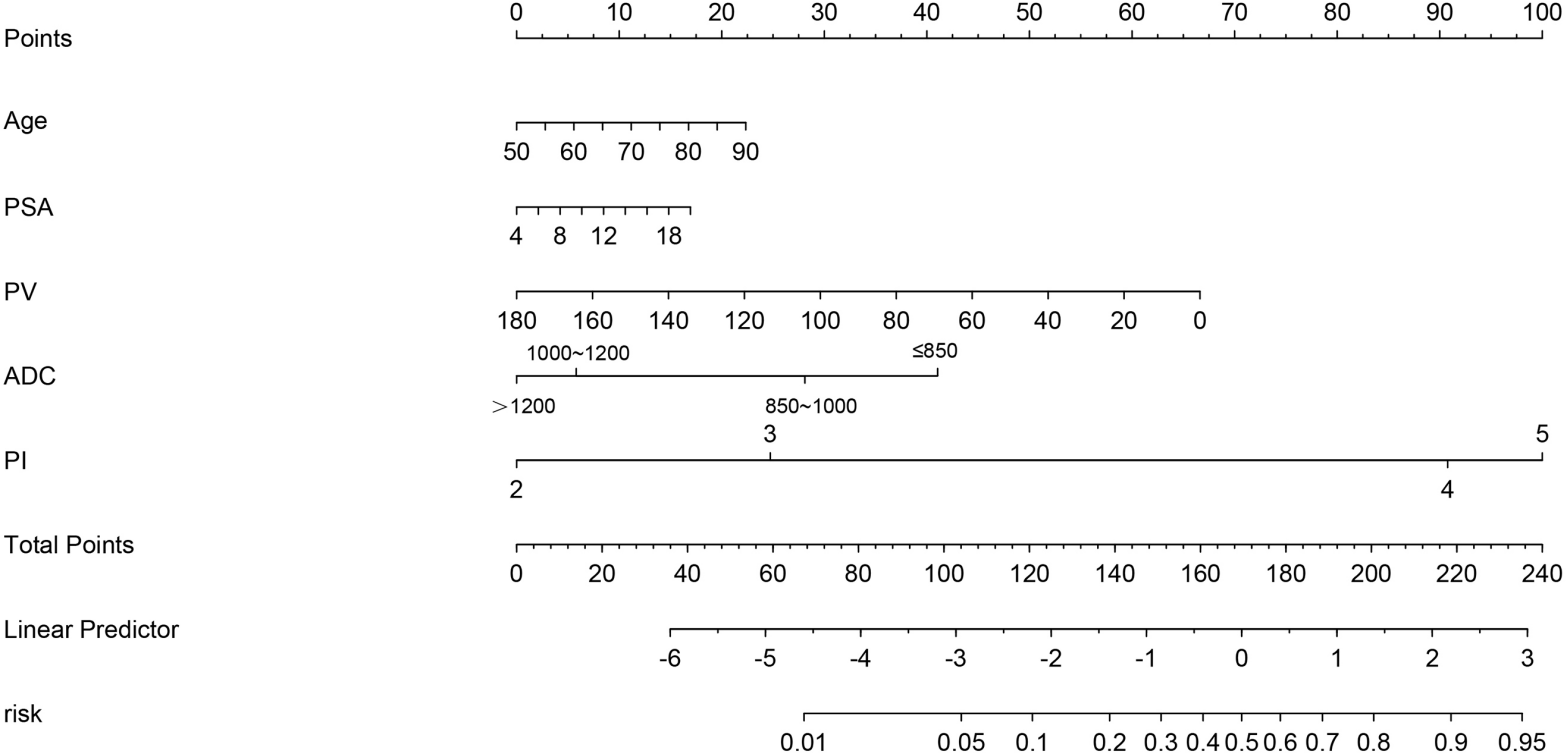
The nomogram integrating the PI-RADS score, ADC value, and clinical characteristics (PSA, PV, and age).

**Figure 4:**
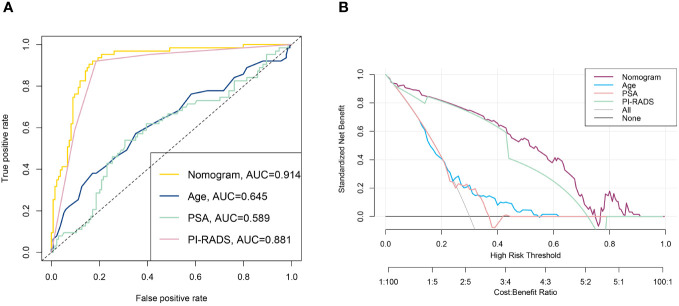
The ROC curve **(A)** and DCA curve **(B)** of the nomogram.

Nomograms are applied diffusely as statistical diagnostic and prognostic models to help clinicians more easily understand the prognosis of tumor patients and improve the diagnosis rate. We constructed a nomogram including PI-RADS score, ADC value, age, PSA, and PV (**Figure 3**). ROC curve analysis showed that the AUC value of the nomogram was 0.944, which was superior to the PI-RADS score (**Figure 4A**). The decision curve analysis also revealed that the nomogram was superior to the PI-RADS score (**Figure 4B**).

## 4 Discussion

In the past few years, multiple multicenter studies (such as PROMIS and PRECISION) recommended the mpMRI before biopsy and the MRI-targeted biopsy for men with clinically suspicious PCa and lesions of suggestive cancer on prostate mpMRI (4, 5, 21). These studies also demonstrated that the MRI-targeted biopsy is superior to the TRUSB in detecting csPCa and avoiding unnecessary biopsy. In our study, we combined the MRI-targeted biopsy and TRUSB together (combined biopsy) to evaluate its diagnostic value in csPCa for biopsy-naïve patients with PSA 4~ 20 ng/ml. We found that the cancer detection rates (CDRs) of PCa and csPCa were 44.7% and 32.0% in the CB group, respectively. Dominik et al. conducted a retrospective study on 745 patients with clinical suspicion of PCa and concluded that the combination of systematic biopsy and MRI-targeted biopsy should be considered in biopsy-naïve patients with PI-RADS scores 3 and 4 (22). Our study found that the CB is superior to TRUSB in detecting csPCa in biopsy-naïve patients with PSA 4~20 ng/ml. Consequently, we propose that the combined biopsy (TRUSB and MRI-targeted biopsy) might be the best fit for biopsy-naïve patients with PI-RADS scores 3, 4, and PSA 4~20 ng/ml.

Prostate-specific antigen (PSA) has been the most commonly utilized indicator of early diagnosis and active surveillance of PCa. Patients with PSA > 4 ng/ml are generally recommended for prostate biopsy. However, when the PSA levels range from 4 to 10 ng/ml, which is often referred to as the “gray zone”, less than 30% of men had positive outcomes of the prostate biopsy, meaning that more than 70% of men will undergo unnecessary prostate biopsy (23, 24). The incidence of PCa and PSA levels varies among different races, and the cancer detection rates of PCa in the Chinese population are much lower than in the European and American populations (25). Therefore, many studies recommend that the PSA level of the “gray zone” in Chinese men should be higher than the referred “gray zone”. Consequently, biopsy-naïve men with PSA 4~20 ng/ml were eventually included in our study, which was consistent with previous studies (25, 26).

This is the first retrospective study to assess the predictive value of ADC, PI-RADS score, PSAd, and the combination of them in PCa and csPCa for biopsy-naïve men with PSA 4~20 ng/ml in China. In our study, men with PCa were older and had higher PSAd levels and PI-RADS, and lower ADC values than men with noncancer. To avoid multicollinearity, the PSAd was incorporated in the establishment of multivariable models, which showed better predictive ability in csPCa than PSA level and prostate volume alone. Thus, we constructed three multivariable models based on the ADC, PI-RADS score, and PSAd: the ADC+PSAd (AP) model, the PI-RADS+PSAd (PP) model, and the PI-RADS+ADC+PSAd (PAP) model. According to the ROC curve analysis, the PAP model had the highest AUCs in predicting the PCa and csPCa for biopsy-naïve men with PSA 4~20 ng/ml, which were 0.930 and 0.913, respectively. When the biopsy risk threshold of csPCa determined by the PAP model was ≥ 0.10, the sensitivity, specificity, PPV, and NPV were 0.95, 0.75, 0.65, and 0.97, respectively. As revealed in the study by Satoshi et al., the PI-RADS score of ≥ 3 yielded a sensitivity of 0.85, a specificity of 0.72, a PPV of 0.75, and an NPV of 0.84 (27). Boesen et al. developed a predictive model based on bpMRI scores and clinical parameters, and they applied the biopsy risk threshold of 20% for csPCa with a sensitivity of 0.93, a specificity of 0.66, a PPV of 0.65, and an NPV of 0.94 (28). In the study of Matteo, they combined the PI-RADS v2 and PSAD to predict the biopsy outcomes and found that patients with a PI-RADS v2 score of < 3 and PSAD < 0.3 ng/mL/mL may avoid the prostate biopsy (29). Therefore, the combination of PI-RADS, ADC values, and PSAd to predict the biopsy outcomes in biopsy-naïve men could improve the performance of PI-RADS and PSAd alone, and when the risk threshold of 0.10, 51% of patients could avoid unnecessary biopsy, and only 5% patients of csPCa were missed.

More and more studies have demonstrated that PSA density (PSAd) could improve the cancer detection rates of PCa compared to PSA alone and serve as an independent predictor for PCa and csPCa (27, 30). The cut-off value of PSAd 0.15 ng/ml^2^ was widely utilized and proven to reduce unnecessary biopsy by combining PI-RADS scores (27, 28, 31). Satoshi et al. propose that men with PSAd < 0.15 ng/ml^2^ and a PI-RADS score ≥ 3 may avoid unnecessary prostate biopsies (27). Moreover, in Boesen’s report, the PSAd 0.15 could reduce 41% of unnecessary prostate biopsies and miss only 5% of men with csPCa (31). However, the PSAd of 0.15 ng/ml^2^ was mainly based on the European and American populations, which may not be suitable for the Chinese population. In our present study, the optimal cut-off value of PSAd in men with PSA 4~20 ng/ml was 0.23 and 0.33 ng/ml^2^ with PI-RADS scores ≥ 2 and ≥ 3, respectively, which were similar to other multicenter studies in China (32, 33).

Based on the multivariate logistic regression analysis, we found that the PI-RADS score and ADC value were all independent predictors of PCa and csPCa. The prostate volume was calculated using the ellipsoid formulation in our study, which is recommended by PI-RADS version 2.1 (10). We also found that men with csPCa had low ADC values in mpMRI compared to men with non-csPCa, and ADC value was also an independent predictor of PCa and csPCa and negatively correlated with Gleason score (*r* = −0.364, *p* < 0.001). Wang et al. also demonstrated that mean ADC shows the strongest correlation with the Gleason score and has the best capability of predicting csPCa (34). A systematic review of 2,457 patients from 39 studies also identified a moderate correlation between ADC value and Gleason score by meta-analysis (35). Dong et al, established MRI-based radiomics models (consisting of ADC value and T2WI) to discriminate between benign and malignant prostate lesions and predict extracapsular extension and positive surgical margins (14). Additionally, the ADC value also has a negative relationship with the proliferation marker Ki 67, which illustrates that ADC is associated with proliferation potential (36).

We must recognize that our study had some limitations. Firstly, our analysis refers to a retrospective study from a single center with a limited number of men, and selection bias is inevitable, which may affect the precision and accuracy of our results. Therefore, multicenter clinical data are needed to validate the practicability of our study. Secondly, Gleason score and pathological results were obtained from the biopsy (combined biopsy and TRUSB) reports rather than pathological slices and reports after radical prostatectomy. Thus, there may be some false-negative results and an underdetection of csPCa. Thirdly, at present, the PSA level, a digital rectal examination (DRE), or both of them have become the primary screening methods for PCa (37). However, our study did not include the DRE due to the subjective and empirical judgment of a surgeon and partial data incompleteness. Finally, we only included biopsy-naïve patients with PSA 4~20 ng/ml in our study, which would affect the universality of our models and analysis. Patients who had undergone prostate biopsy with negative outcomes were excluded from our study, which may also affect the universality of our models and analyses. However, some studies revealed that mpMRI can report a comparable detection rate of csPCa between biopsy-naïve patients and patients who had undergone prostate biopsy with negative outcomes (38), which may demonstrate that whether or not the biopsy was performed before did not affect the mpMRI results. Nevertheless, this is one of our strengths in combining PSAd and mpMRI-based radiomics to forecast biopsy outcomes in men with PSA 4~20 ng/ml, which may be the “grey zone” in Chinese men.

## 5 Conclusion

For biopsy-naïve patients with PSA 4~20 ng/ml, the combined biopsy of the prostate (TB+SB) is superior to TRUS-SB in detecting csPCa and reducing icsPCa. The multivariable model based on the mpMRI-based scores and PSAd could provide a reference for clinicians in forecasting biopsy outcomes in biopsy-naïve patients with PSA level 4~20 ng/ml and make a more comprehensive assessment during the decision-making of the prostate biopsy.

## Data availability statement

The raw data supporting the conclusions of this article will be made available by the authors, without undue reservation.

## Ethics statement

This study was approved by the ethics committee of the First Affiliated Hospital of Sun Yat-sen University (2023-439), which waved off the requirement for informed consent. This study conformed to the ethical guidelines of the 1975 Declaration of Helsinki.

## Author contributions

LR: Conceptualization, Data curation, Formal analysis, Investigation, Methodology, Software, Writing – original draft, Writing – review & editing. YC: Conceptualization, Data curation, Formal analysis, Investigation, Writing – review & editing. ZL: Conceptualization, Data curation, Formal analysis, Methodology, Writing – review & editing. GH: Conceptualization, Data curation, Formal analysis, Investigation, Writing – review & editing. WW: Conceptualization, Data curation, Formal analysis, Writing – review & editing. XY: Conceptualization, Data curation, Writing – review & editing. BB: Conceptualization, Data curation, Writing – review & editing. YG: Conceptualization, Data curation, Writing – review & editing. JL: Conceptualization, Data curation, Formal analysis, Investigation, Writing – review & editing. XM: Conceptualization, Data curation, Formal analysis, Supervision, Visualization, Writing – review & editing, Funding acquisition.

## Supplementary material

Supplementary Figure 1 Number and proportion of patients stratified by PSAd, ADC value, and PI-RADS scores.
