# Supplementary material for: Integration of PSAd and multiparametric MRI to forecast biopsy outcomes in biopsy-naïve patients with PSA 4~20 ng/ml
Source: Front Oncol. 2024 Jul 4;14:1413953. doi: 10.3389/fonc.2024.1413953 (PMC11254766; doi:10.3389/fonc.2024.1413953)
Supplement: Supplementary file 1 [file Table_1.docx]

**Table S1** Clinical features of PCa patients with prostate mpMRI.

| **Features** | **csPCa** | **non-csPCa** | **P value** |
| --- | --- | --- | --- |
| Age, years *  PSA, ng/ml * | 70.1 (66.0-76.0)  12.11 (7.66-15.73) | 66.9 (63.0-71.0)  10.71 (7.58-13.79) | 0.004  0.029 |
| Prostate volume, cm^3^ * | 37.4 (26.0-42.6) | 55.5 (34.6-69.3) | <0.001 |
| PSAd, ng/ml^2^ *  mpMRI-based radiomics | 0.39 (0.23-0.51) | 0.23 (0.14-0.29) | <0.001 |
| PI-RADS (v2.0) † |  |  | <0.001 |
| 2 | 3 | 79 |  |
| 3 | 2 | 30 |  |
| 4 | 21 | 12 |  |
| 5 | 37 | 13 |  |
| ADC value * | 893 (663-913) | 1223(1034-1298) | 0.098 |

* Data are referring to medians, with interquartile ranges in parentheses (IQR).

† Data are referring to numbers of patients.

**Table S2** Spearman correlation between clinical features/mpMRI data

and Gleason scores.

| **Features** | **R** | **P value** |
| --- | --- | --- |
| Age  PSA | 0.080  0.074 | 0.461  0.495 |
| Prostate volume | 0.035 | 0.749 |
| PSAd  mpMRI-based radiomics | 0.051 | 0.634 |
| PI-RADS (v2.0) | 0.479 | <0.001 |
| ADC value * | ‒0.364 | <0.001 |

**R**, correlation coefficient

**Table S3** The avoided biopsy and missed csPCa of different biopsy strategies and their sensitivity, specificity, and predictive values (positive and negative) in biopsy-naïve patients with PSA 4 ~ 20 ng/ml.

|  | Avoided biopsy | Missed csPCa | **Sensitivity** | **Specificity** | **PPV** | **NPV** |
| --- | --- | --- | --- | --- | --- | --- |
| **PAP model** |  |  |  |  |  |  |
| ≥0.04  ≥0.10  ≥0.15  **PI-RADS**  ≥3  ≥4 | 85(43%)  101(51%)  107(54%)  82(42%)  114(58%) | 2(3%)  3(5%)  4(6%)  3(5%)  5(8%) | 0.97  0.95  0.94  0.95  0.92 | 0.63  0.75  0.80  0.59  0.81 | 0.55  0.65  0.69  0.52  0.70 | 0.98  0.97  0.96  0.96  0.96 |

PPV, positive predictive value; NPV, negative predictive value.
