# Supplementary figures and images for: Integration of PSAd and multiparametric MRI to forecast biopsy outcomes in biopsy-naïve patients with PSA 4~20 ng/ml
Source: Front Oncol. 2024 Jul 4;14:1413953. doi: 10.3389/fonc.2024.1413953 (PMC11254766; doi:10.3389/fonc.2024.1413953)

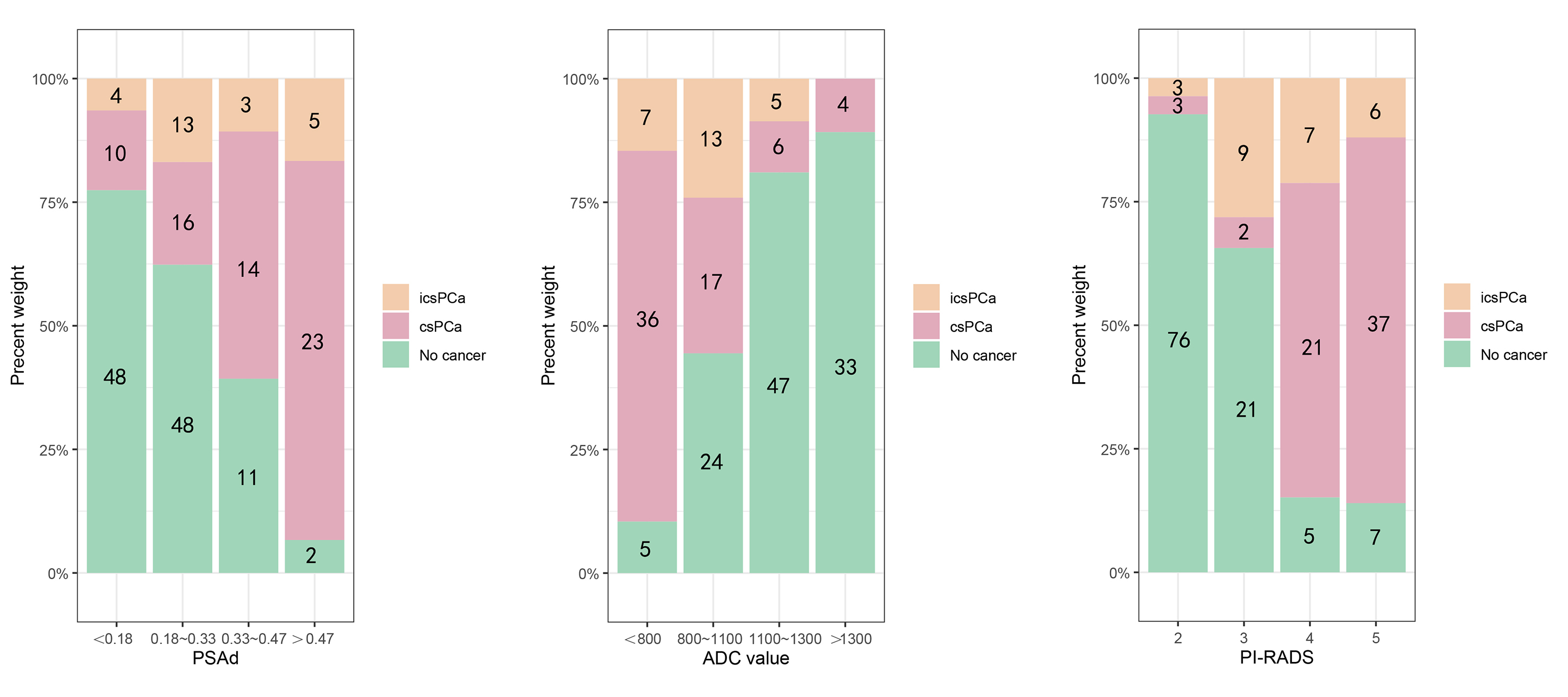

Supplement: Supplementary Figure 1 — Number and proportion of patients stratified by PSAd, ADC value, and PI-RADS scores. [file Image_1.jpeg]
